# Pesticide Product Use and Risk of Non-Hodgkin Lymphoma in Women

**DOI:** 10.1289/ehp.7070

**Published:** 2004-06-03

**Authors:** Ikuko Kato, Hiroko Watanabe-Meserve, Karen L. Koenig, Mark S. Baptiste, Patricia P. Lillquist, Glauco Frizzera, Jerome S. Burke, Miriam Moseson, Roy E. Shore

**Affiliations:** ^1^Department of Environmental Medicine, New York University of School of Medicine, New York, New York, USA; ^2^Bureau of Chronic Disease Epidemiology and Surveillance, New York State Department of Health, Albany, New York, USA; ^3^Department of Pathology, New York University Medical Center, New York, New York, USA; ^4^Department of Pathology, Alta Bates Summit Medical Center, Berkeley, California, USA

**Keywords:** case–control study, mothballs, NHL, pesticides

## Abstract

A population-based, incidence case–control study was conducted among women in upstate New York to determine whether pesticide exposure is associated with an increase in risk of non-Hodgkin lymphoma (NHL) among women. The study involved 376 cases of NHL identified through the State Cancer Registry and 463 controls selected from the Medicare beneficiary files and state driver’s license records. Information about history of farm work, history of other jobs associated with pesticide exposure, use of common household pesticide products, and potential confounding variables was obtained by telephone interview. Odds ratios (ORs) and 95% confidence intervals (CIs) were estimated using an unconditional logistic regression model. The risk of NHL was doubled (OR = 2.12; 95% CI, 1.21–3.71) among women who worked for at least 10 years at a farm where pesticides were reportedly used. When both farming and other types of jobs associated with pesticide exposure were combined, there was a progressive increase in risk of NHL with increasing duration of such work (*p* = 0.005). Overall cumulative frequency of use of household pesticide products was positively associated with risk of NHL (*p* = 0.004), which was most pronounced when they were applied by subjects themselves. When exposure was analyzed by type of products used, a significant association was observed for mothballs. The associations with both occupational and household pesticides were particularly elevated if exposure started in 1950–1969 and for high-grade NHL. Although the results of this case–control study suggest that exposure to pesticide products may be associated with an increased risk of NHL among women, methodologic limitations related to selection and recall bias suggest caution in inferring causation.

The U.S. Environmental Protection Agency (EPA) estimates that approximately 1.2 billion pounds of pesticides were used in the United States in 1999 (Donaldson et al. 2002), which was equivalent to 4.4 pounds per capita in the U.S. population. Of these pesticides, 76% were used in agriculture, 11% in other industries/governments, and 13% in homes and gardens; also, they were used by 77% of U.S. households and 1.2 million certified professional applicators (Donaldson et al. 2002). Despite a recent decline in overall usage after a marked increase in the 1950s and 1960s, and despite the fact that registrations of some pesticides found to have unacceptable toxicity have been canceled, there has been a concern about their long-term effects on human health, because some pesticides persist in human tissues, soil, foods, and the home environment (Muller 2000).

One of the major health concerns is carcinogenicity. More than 30 pesticides or groups of pesticides have been identified as possible carcinogens to humans by several national and international institutions [International Agency for Research on Cancer (IARC) 1987, 1991; U.S. EPA 2004]. Pesticides may increase the risk of cancer through various mechanisms. Some are known to be genotoxic (mutagenic) or tumor promotive, whereas others possess hormonal, immunotoxic, or hematotoxic properties (Acquavella et al. 2003; Dich et al. 1997). Furthermore, it has been reported that exposure to certain pesticides synergistically increases the mutagenicity of diet-derived heterocyclic amines (Wagner et al. 2003). Higher frequencies of chromosome aberrations, sister chromatid exchanges, and micronuclei have been observed in peripheral lymphocytes of pesticide applicators and certain groups of farmers (Bolognesi 2003; Maroni and Fait 1993). Because of these chromosome abnormalities, cancers in the hematolymphoid tissues [e.g., non-Hodgkin lymphoma (NHL), Hodgkin lymphoma, multiple myeloma, and leukemia] have been a central issue in the evaluation for potential health consequences of pesticide exposure. Particularly, NHL has received research attention because the recent rapid increase in its incidence parallels an exponential growth in pesticide use with a few decades of lag (Ries et al. 2003).

There have been extensive reviews (Acquavella et al. 1998; Dich et al. 1997; Maroni and Fait 1993; Morrison et al. 1992; Zahm and Ward 1998) on cancer risk associated with farming and pesticide exposure as well as a number of more recent articles on specific types of cancer and specific classes of pesticides (Blair et al. 1998; Buckley et al. 2000; Cantor et al. 2003; Hardell et al. 2002; Kogevinas et al. 1995; McDuffie et al. 2001; Meinert et al. 2000; Nanni et al. 1996; Schroeder et al. 2001; Waddell et al. 2001; Woods et al. 1987; Zahm et al. 1990; Zheng et al. 2001). However, the vast majority of those studies have focused only on occupational exposures, except for some childhood cancer studies in which parental exposures in and around the home were assessed (Buckley et al. 2000; Meinert et al. 2000; Zahm and Ward 1998). Because of the widespread use of these chemicals in and around the home and because of the longer time spent at home than at work, especially among women, information about pesticide use around the home is critical to obtain a better picture of the overall effects of pesticides in the general population. In this population-based case–control study in upstate New York, we attempted to address whether pesticide product use at home as well as at work is associated with increased risk of NHL among women.

## Materials and Methods

### Study population.

This study was designed as a population-based case–control study of incident NHL in the upstate counties of New York State (NYS; i.e., excluding New York City and surrounding counties) to examine the associations with several environmental exposures. The study population base consisted of women 20–79 years of age who lived in the defined area of NYS at any time during the case-ascertainment period. Males were excluded because a primary focus of the study was on hair dyes, which will be reported separately. Women with a prior history of any type of hematologic cancer were also excluded from the study population.

Cases were newly diagnosed with NHL during the 3-year period between 1 October 1995 and 30 September 1998 and were identified through a rapid case-ascertainment system coordinated with the NYS Cancer Registry. The completeness of case ascertainment was verified by linkages with the whole state cancer registry database and with state death certificates. From 722 initially identified eligible cases, we excluded 3.4% because their physician’s consent could not be obtained and an additional 4.2% because we could not find a valid contact address of the patients. Population-based controls were frequency matched to the projected age distribution of the cases and were selected from an age-stratified random sample from the NYS Department of Motor Vehicles (DMV) driver’s license files for those < 65 years of age, or from the Health Care Financing Administration (HCFA) beneficiary files for those ≥ 65 years of age. However, the frequency matching was only partially successful because of age-related differences in response rates. To increase comparability between cases and controls, we excluded cases < 65 years of age without a valid NYS driver’s license. No monetary incentives were offered for participation. Among those with valid address information who met all other eligibility criteria, the final participation rate in the study was 56% (*n* = 376, with a median age at diagnosis of 65 years) among the cases, 30% (*n* = 248) among the DMV controls, and 67% (*n* = 215) among the HCFA controls. The participation rate of cases and DMV controls was low in part because of a requirement by the NYS Department of Health institutional review board that they first be sent a study solicitation letter by the NYS Cancer Registry; only if they returned a signed consent form could we contact them for an interview. Verbal consent to participate in the study was approved by the New York University (NYU) School of Medicine institutional review board for the HCFA controls.

Demographic characteristics of the participants have been published elsewhere (Kato et al. 2002). Briefly, both the case and control participants were primarily white (98%), born in NYS (77%), and married (59%). Mean age at the index date (defined below) was 60.5 years for the cases and 54.6 years for the controls. More controls had a college education (61%) than did cases (45%). The proportion of smokers was similar in the two groups (22% in cases and 19% in controls). Family history of hematologic cancer was more common in cases (11%) than in controls (6%).

### Data collection.

Cases and controls were interviewed over the telephone by an interviewer at NYU who was not aware of the case–control status of the participants. The structured questionnaire was developed specifically for this study. Next of kin were interviewed for the cases (20.5%) and controls (3.2%) who were found to be deceased or medically incapable of participating in an interview. The most common surrogates were children (47%), followed by husbands (27%). In advance of the interview, each participant was mailed a package containing a letter outlining the study and a booklet displaying lists of product/chemical names to be discussed in the interview. The median time between NHL diagnosis and the telephone interview was 1.2 years, ranging from 2 months to 3.3 years. Information was collected on the lifetime history of living or working on a farm, exposures to pesticides from other types of jobs, and the lifetime history of pesticide product use in and around the home. For the subjects who worked on a farm, we asked whether pesticides were used on the farm and whether the pesticides were applied by the subject herself. When the subject applied or handled pesticides herself, details about pesticides (name and duration) were elicited. We asked about other occupational exposures in three separate categories: insecticides, herbicides, and wood preservatives. For each category, the number of hours exposed per day, week, month, or year and total duration of employment were elicited. We asked about pesticide product use in and around the home in 12 separate categories principally based on the purposes of use: to control ants, cockroaches/silverfish, bees, flies/mosquitoes, moths (mothballs), or termites; to treat indoor plants, trees/shrubs, plants in the garden/outdoor pots, or lawns; to control head lice; and use of an indoor/outdoor fogger. For each group of pesticide products, information on application methods (indoor/outdoor and by self/others), year or age first used, year or age last used, and average frequency of use in a year/season was elicited. Based on the average frequency and total duration of use, we calculated the cumulative number of uses for each product or group of products as well as for each mode of application.

### Classification of NHL.

Copies of medical records of the cases were obtained and reviewed to confirm their diagnosis and eligibility. In addition, to allow for a uniform classification of NHL, pathology slides were obtained and reviewed by an expert hematopathologist at NYU (G.F.). It was possible to complete the review for 268 cases (71%). Approximately 26% of these slides were sent to a second expert hematopathologist consultant (J.S.B.) to resolve discrepancies between the original diagnoses and the review diagnoses at NYU. In our review, NHL was classified according to both the REAL (Revised European-American Classification of Lymphoid Neoplasms) system (Harris et al. 1994) and the Working Formulation (Weisenburger 1992). Classification by immunophenotype was based on the final REAL categories from our pathologic review whenever available, otherwise on the immunophenotype obtained at the original institution. If neither was available (9.8%), follicular lymphomas by histology were considered B-cell in type, and the others were left unclassified. As a result, 322 were considered B-cell, 25 T-cell, and 29 unclassified. Lymphomas were also grouped by grade based on the Working Formulation: 54 low grade, 189 intermediate grade, 25 high grade, and 8 unclassified.

### Statistical analysis.

In order to eliminate reported exposures that occurred after diagnosis of NHL and to allow a minimum latency (lag) period of 1 year from exposure to diagnosis for each case, we set an index date, after which any exposures should be excluded from the analysis. The index date was defined as the date 1 year before diagnosis. To ensure comparable exposure assessment periods between cases and controls, within 5-year age strata we randomly assigned lag periods (i.e., index dates) to controls corresponding to the frequency distribution of lags among the cases of comparable age. Any exposures and events reported after their index dates were discounted for both cases and controls. The average lag time between the index date and the date of interview was 2.5 years for both cases and controls.

The odds ratios (ORs) and 95% confidence intervals (95% CIs) for NHL according to various indices for pesticide exposure were calculated using the unconditional logistic regression model (Breslow and Day 1980), adjusted for selected covariates: four continuous variables (age at index date, year of interview, and frequencies of use of pain-relieving drugs and of cortisone injections) and five indicator variables (college education, surrogate interview, family history of hematologic cancer, and personal history of eczema/hives and of antihistamine use). These covariates were selected according to the following three criteria: *a*) known risk factors for NHL (age and family history of hematologic cancer); *b*) factors that influence data quality (education, surrogate status, and year of interview); and *c*) potential risk factors associated with pesticide product use/farm work (frequencies of use of pain-relieving drugs and of cortisone injections and personal history of eczema/hives and of antihistamine use) (Holly et al. 1999; Kato et al. 2002; McWhorter 1988). Whenever possible, the ORs were calculated for ordered categories (in quartiles, tertiles, or halves) of cumulative number of uses or total duration of exposure, compared with nonusers or no-exposure groups. Tests for linear trend in the logit of risk with increasing frequency or duration of exposure were performed using natural-log–transformed continuous values. Selected analyses were repeated for subtypes of lymphoma. All statistical analyses were conducted using SAS software (SAS Institute, Cary, NC).

## Results

First, we examined the associations with potential exposure to pesticides at work (Table 1). There was a marginal trend in risk of NHL with the number of years worked on a farm (*p* = 0.053). This trend became more significant (*p* = 0.03) when only farm work involving pesticide use was considered. The OR associated with such farm work of ≥ 10 years was 2.12 (95% CI, 1.21–3.71). Applying or handling pesticides by the women themselves was not associated with appreciably increased risk. Furthermore, < 50% of the women who applied/handled pesticides could recall the product names; thus, evaluation by chemical class of pesticides was not feasible. When types of crops handled by the study subjects were considered, the OR appeared to be higher for vegetables, grain, and other crops than for fruits and flowers, although none of them was statistically significant. Exposure to pesticides was also reported under various types jobs other than farming (*n* = 61). About half of these jobs (*n* = 32) involved a passive low level of exposure to periodic building/lawn treatment with pesticides. Common jobs in this category were restaurant jobs, office work, and miscellaneous other jobs. The second category of jobs (*n* = 9) represented a possible intermediate level of exposure, for example, retail jobs handling pesticides, crop-processing factory work, or working in an office adjacent to a farm or florist. The final category of jobs represented occupations that may have entailed direct exposure to pesticides through application (*n* = 20). This consisted of structure maintenance or environmental control jobs, horticultural work, veterinary jobs, and wood-handling factory jobs. The number of hours of actual exposure was reported to be much shorter for the low-exposure job category (median, 12 hr/year), compared with those in the intermediate- and high-exposure job categories (medians, 192 hr/year and 55 hr/year, respectively). With increasing cumulative number of hours exposed to pesticides at these jobs other than farming, there was a marginal increasing trend in risk of NHL (*p* = 0.08). When farming and other jobs associated with pesticide exposure were combined, the total duration at any of these jobs was significantly positively associated with the risk of NHL (*p* = 0.005). This increase in risk of NHL was more pronounced when exposure started in 1950–1969 than when it first occurred before or after this period.

The ORs and 95% CIs associated with pesticide use in and around the home are presented in Table 2. We grouped products based on the target pest. As a result, insecticides were categorized into those for crawling insects (ants, cockroaches/silverfish, and termites), for flying insects [bees, flies/mosquitoes, and moths (except mothballs) and indoor/outdoor fogger], mothballs, and antilice products. Products to treat indoor plants, trees/shrubs, or plants in garden/outdoor pots were combined into one group, that is, fungicides/plant pesticides. Products to treat lawns were considered herbicides/lawn pesticides. Products to control moths were assumed to be mothballs if they were used exclusively indoors; otherwise, they were counted in the categories for the flying insects. Correlations among these groups of home pesticide products ranged from –0.07 to 0.27. For all products combined, there was a linear increase in risk of NHL with increasing cumulative number of uses (*p* = 0.004). The positive trend was observed for most of the products groups, except for the herbicide and fungicide groups. Logistic regression for individual product groups with simultaneous adjustment for use of all other products revealed a significant positive association of NHL with mothballs (*p* = 0.03) and a marginally significant association with insecticides for flying insects/foggers (*p* = 0.07). When no-exposure groups were excluded from the trend analyses, the regression coefficient for mothballs approached zero, whereas those for the others changed minimally. When time of first use was analyzed for all household pesticide products combined, the association with NHL was significant only for pesticide use started during 1950–1969 (OR = 2.42; 95% CI, 1.16–5.02), whereas weaker associations were found for pesticide use started before 1950 or after 1969 (OR = 1.42 and 1.25, respectively; data not shown).

For pesticides for flying and crawling insects and for all pesticide products combined, we calculated the ORs for NHL according to application methods that were separated into three groups based on presumed exposure intensity, namely, pesticides applied by the respondent, applied indoors by others, or applied outdoors by others (Table 3). For individual groups of pesticide products, we also adjusted for other pesticide use via the same application method in these analyses. The positive linear trend with cumulative number of uses was most evident when pesticides were applied by women themselves for all products combined (*p* = 0.01), but the risk associated with insecticides for flying insects was only significant when they were applied outdoors by others. The association with mothballs was virtually the same when exposure occurred through self use or use by others, although a limited number of subjects were exposed through use by others (data not shown).

We also examined combined and separate effects of occupational and home pesticide exposure. To study combination effects, we divided exposures into two levels using the medians: 10 years for duration of jobs associated with pesticide exposure and 70 times for cumulative number of uses of any household pesticide products. The OR was 2.33 (95% CI, 0.93–5.85) for the subjects with higher exposures for both (*n* = 54), 1.46 (95% CI, 0.72–2.98) for those with higher exposure only at home or only at job (*n* = 381), and 1.00 (95% CI, 0.49–2.04) for those who had lower exposure for both or combinations of no exposure and lower exposure at home and job (*n* = 354), compared with the subjects with neither exposure (*n* = 48), and this trend was statistically significant (*p* = 0.005). When the subjects were limited to those without any occupational exposure to pesticides (*n* = 648), the association with cumulative number of uses of any type of home pesticide products remained highly statistically significant (*p* = 0.005). The number of women who were not exposed to any home pesticide products was too small (*n* = 54) to analyze the effects of occupational exposure separately. However, simultaneous adjustment for home pesticide use did not affect the association with occupational pesticide exposure (*p* = 0.01).

Table 4 presents the results of analysis by subtype of NHL according to levels of total pesticide exposure from work and around the home. There were no clear differences in trends in the ORs between B-cell and T-cell subtypes, but the increasing risk of NHL with the number of years worked in pesticide-related jobs and with the cumulative number of any pesticide product uses around the home was most pronounced for high-grade lymphoma (*p* < 0.001 and *p* = 0.002, respectively).

## Discussion

The results of this case–control study suggest that exposure to pesticide products may lead to an increased risk of NHL among women. This finding was supported by the dose–response relationship observed with length of exposure, cumulative number of uses, and potential intensity of exposure.

Compared with studies using biologic or environmental samples at single time points, a questionnaire-based study has an advantage in the assessment of long-term exposure by reconstructing the whole personal history. However, it also has limitations. First, there may be bias in recall: cases with serious disease may be likely to report hypothesized exposures more completely than controls in good health. This especially may occur when there is enhanced public health concern about an exposure (Infante-Rivard and Jacques 2000; Weinstock et al. 1991), as may be the case for pesticides.

Obtaining information on specific chemicals over a long period of time is challenging, given the large number of products on the market, but is crucial when exposure effects may be cumulative. For nonoccupational exposure, Teitelbaum (2002) has suggested that asking about treatments for specific pest problems may be an effective way to help subject recall, a practice we implemented in designing our questionnaires. Notably, reasonable correlations have been observed between self-reported household chemical use and measurements of pesticides and their metabolites in urine of household members (Kieszak et al. 2002) and in indoor air (Van Winkel and Scheff 2001). Therefore, this type of questionnaire design seems useful in the assessment of household pesticides, at least for recent exposure. One shortcoming of our assessment of nonoccupational pesticide exposure is that we did not include dietary exposure, which may contribute a substantial fraction of pesticide exposures (Whitmore et al. 1994; Yess et al. 1991). However, mis-classification of exposure due to the omission of dietary sources is most likely to be nondifferential because many foods are known to contain pesticide residues (Yess et al. 1991).

It has been suggested that self-reported occupational pesticide exposure tends to overestimate exposure (Daniels et al. 2001; Meinert et al. 2000) because people often do not know for sure about actual chemical contents used at their work places. Farmers may be an exception (Blair and Zahm 1990), but indeed fewer than half of the women who applied pesticide themselves in this study could recall at least one of the product names they used. This proportion appears to be lower than in farmer studies (Dosemeci et al. 2002; Zahm et al. 1993) but may be because most of the farm work was in the distant past (median interval between last farm work and interview was 37 years, and median duration of farm work was only 8 years). Poor recall may also account for our failure to detect an excess risk among women who applied or handled pesticides. However, reentry to areas that were recently treated with pesticides for harvesting may result in greater cumulative exposure to pesticide residues than application itself (Garcia 2003); Coronado et al. (2004) recently reported that detectable levels of pesticide metabolite were not higher among workers who were engaged in mixing, loading, or applying pesticide formulations than among those who did not perform these tasks, contrary to expectation. Some investigators have found that including information from surrogates biases the results (Blair and Zahm 1990; Waddell et al. 2001), but when we limited our analysis to the subjects themselves, the strength of the associations remained almost the same as those observed in the entire sample.

Finally, the relatively low overall participation rate in this study raises issues of selection bias and of generalizability of the results. The probable reasons for the lower response rates among the DMV controls and the cases have been discussed elsewhere (Kato et al. 2002). Cases in this study were similar in age distribution to all the cases diagnosed in NYS during the same time period, but white and married women were overrepresented in both the case and control groups. Although we do not have external data to estimate the magnitude of selection bias, the results of hypothetical sensitivity analyses based on a selection bias factor defined by Rothman and Greenland (1998) suggest that the ORs obtained in this study are more likely to have been underestimated than overestimated. This relies on an assumption that exposed controls were more likely to respond to this survey than were nonexposed controls because both the study invitation letter and the study packet (product list) indicated that pesticides were one of our major research interests, whereas this selection should play a minor role among the cases who were already motivated because of their diagnosed disease. In addition, the DMV controls, who were < 65 years of age and had a lower overall participation rate than cases, may have been more motivated to participate in research related to environmental issues and therefore may have had better recall of pesticide exposure. This would tend to counterbalance the hypothesized biased recall among cases discussed above, unless such motivated people tend to live in better housing conditions that require less use of pesticides.

It is possible that pesticide use is a marker for other possible causative factors for NHL. For instance, occupational exposure to pesticides is often accompanied by exposure to other possible hazardous substances, such as solvents, fuels, and dusts (Maroni and Fait 1993; Morrison et al. 1992), that have been associated with increased NHL risk (Mao et al. 2000; Rego 1998). Similarly, people who use pesticides in and around the home may tend to use other household chemicals more often than those who do not. Another possibility is that pesticide use is an indicator of exposure to insects that may act as vectors to transmit viruses and bacteria. Certain types of viruses and bacteria have been identified as etiologic factors for NHL (Pagano 2002; Persing and Prendergast 1999).

Some earlier studies have pointed to associations between specific types of pesticide or pesticide groups and NHL risk (Dich et al. 1997). Three groups of pesticides have received special research attention: phenoxy herbicides and organochlorine and organophosphate insecticides. However, the results have been inconclusive because initial positive findings that were usually based on small numbers of subjects have often not been confirmed in larger studies or in multivariate analyses taking other pesticides into consideration (Cantor et al. 2003; Hardell et al. 2002; Morrison et al. 1992). In this study, we were not able to analyze any specific classes of chemicals because the women had limited recall of the particular chemicals used. Yet, the finding that pesticide use starting in 1950–1969 was associated with the most pronounced risk of NHL suggests a potential role of organochlorine insecticides that became widely available during this period. Alternatively, it may be a chance finding or simply indicate that a 25–45 year latency period is typical of pesticide-induced NHL.

A finding that is relatively unique in this study is the increased risk of NHL associated with mothball use, although a dose response was not clearly demonstrated among users. In the United States, major chemical constituents of mothballs are naphthalene or *para-*dichlorobenzene (*p*-DCB). These chemicals are also constituents of other common household products, such as air fresheners and solid toilet bowl deodorizers, which were not included in our questionnaire. Vapors from mothballs can be absorbed not only by inhalation but also by direct skin contact. Both of these chemicals are known to have hematotoxicity, including reports of hemolytic anemia (Hallowell 1959; Santucci and Shah 2000) and aplastic anemia (Harden and Baetjer 1978). In addition, *in vitro* and *in vivo* studies have demonstrated cytotoxicity and genotoxicity of these chemicals and their metabolites (Bagchi et al. 1998; Brusick 1986; Carbonell et al. 1991; Tingle et al. 1993), and carcinogenicity has been shown in animal models (Preuss et al. 2003; Umemura et al. 1992). Importantly, both naphthalene and *p*-DCB are among the most ubiquitously detected hazardous household chemicals in indoor air (Van Winkel and Scheff 2001), and concentrations in indoor air samples and urine samples of residents are correlated with reported mothball use (Kieszak et al. 2002; Van Winkel and Scheff 2001). This suggests that the association between mothball use and NHL merits further investigation.

We found that the association with pesticide exposure was most pronounced for high-grade lymphoma. The results for subtypes of NHL, however, should be interpreted cautiously because of small numbers of cases by subtype and because of the multiple comparisons involved. Data have been limited and inconsistent in earlier studies concerning types of lymphoma associated with pesticide exposure. There have been reports of relatively stronger associations of various types of agricultural insecticides with low-grade lymphoma (Nanni et al. 1996), carbamate insecticides with small lymphocytic lymphoma (Zheng et al. 2001), organophosphate pesticides and phenoxy herbicides with intermediate grade lymphoma (Waddell et al. 2001; Zahm et al. 1990), and phenoxy herbicides with B-cell lymphoma (Zahm et al. 1990). Schroeder et al. (2001) reported that a type of B-cell lymphoma that carries a specific chromosomal translocation was associated with occupational exposure to several types of pesticides. Finally, a case–control study of NHL among children revealed that the associations with parental occupational and household exposure to pesticides were more clear for higher grade lymphomas, whereas there were no differences between B- and T-cell types (Buckley et al. 2000). Although mechanistic bases for possible carcinogenic actions by pesticides are largely unknown, Schroeder et al. (2001) speculate that they are different from those for NHL linked to immunosuppression, based on their observation of a specific genetic change associated with pesticide exposure.

In conclusion, the results of our case–control study suggest an association of pesticide exposures with NHL. However, methodologic limitations related to selection and recall bias suggest caution in inferring causation. In order to draw more definitive conclusions and to make public recommendations, more research is needed, integrating various types of studies, such as surveillance for personal pesticide product use, development and application of new biomarkers for pesticide exposure, and assessment of genetic polymorphisms related to pesticide metabolism.

## Figures and Tables

**Table 1:** ORs and 95% CIs for NHL associated with occupational pesticide exposures.

Type of exposure	No. of cases/controls	OR*^a^*	95% CI
Worked on a farm (years)			
0	258/352	1.00	—
0.1–4	26/35	1.03	0.56–1.90
4.1–8	25/28	1.33	0.71–2.48
8.1–15	32/19	2.16	1.09–4.26
≥ 15.1	27/28	1.40	0.74–2.63
		*p**^b^* = 0.053	
Worked on a farm using pesticides (years)*^c^*			
< 10	30/35	1.09	0.61–1.95
≥ 10	43/32	2.12	1.21–3.71
		*p* = 0.020	
Applied pesticides on a farm*^c^*			
Yes	25/24	1.18	0.59–2.38
Crops handled*^c^*			
Fruit	30/35	1.18	0.65–2.13
Vegetables	62/55	1.50	0.96–2.35
Grain	40/33	1.53	0.87–2.69
Other	18/17	1.74	0.79–3.82
Other occupations with pesticide exposure (cumulative hours)			
0	346/432	1.00	—
< 180	13/18	1.11	0.50–2.49
≥ 180	17/13	2.21	0.94–5.17
		*p* = 0.077	
Any occupations with pesticide exposure (years)			
0	277/371	1.00	—
0.1–4.9	16/26	1.01	0.48–2.11
5.0–9.9	22/25	1.13	0.58–2.20
10–17.9	29/20	2.72	1.37–5.40
≥ 18.0	28/20	1.80	0.93–3.48
		*p* = 0.005	
Year of starting job with pesticide exposure			
None	277/371	1.00	—
≤ 1949	39/35	1.24	0.71–2.16
1950–1969	32/21	2.86	1.50–5.45
1970–index date	23/35	1.19	0.63–2.26

a Adjusted for age at index date, family history of hematologic cancer, college education, surrogate status and year of interview, frequencies of use of pain-relieving drugs and of cortisone injections, history of eczema/hives, and history of antihistamine use.

b *p*-Values for trend based on natural-log–transformed continuous values.

c Compared with subjects who never worked on a farm.

**Table 2:** ORs and 95% CIs for NHL associated with home pesticide use.

Type of home pesticides	Cumulative no. of uses	No. of cases/controls	OR*^a^*	95% CI
Insecticides for flying bugs or foggers	0	117/161	1.00	—
	1–3	54/95	0.90	0.56–1.45
	4–16	53/78	1.07	0.66–1.75
	17–86	75/66	1.69	1.04–2.75
	≥ 87	77/63	1.31	0.80–2.15
			*p**^b^* = 0.070	
Insecticides for crawling bugs	0	124/171	1.00	—
	1–3	63/81	1.16	0.73–1.83
	4–15	51/77	0.76	0.46–1.24
	16–46	71/65	1.40	0.86–2.28
	≥ 47	67/69	1.18	0.73–1.92
			*p* = 0.227	
Anti-lice products	0	229/307	1.00	—
	1	56/71	1.20	0.76–1.89
	2–3	45/37	1.48	0.87–2.52
	≥ 4	36/37	1.23	0.69–2.18
			*p* = 0.224	
Mothballs	0	217/354	1.00	—
	1–10	39/24	2.19	1.21–3.97
	11–25	34/32	1.36	0.77–2.42
	26–44	38/27	1.82	1.01–3.29
	≥ 45	39/25	1.33	0.70–2.52
			*p* = 0.025	
Herbicides/lawn pesticides	0	231/287	1.00	—
	1–4	33/44	0.88	0.50–1.53
	5–17	30/47	0.74	0.42–1.32
	18–39	27/41	0.98	0.56–1.71
	≥ 40	40/37	0.89	0.51–1.54
			*p* = 0.658	
Fungicides/plant pesticides	0	201/263	1.00	—
	1–7	35/58	1.01	0.60–1.71
	8–27	36/58	0.80	0.48–1.34
	28–79	52/42	1.42	0.85–2.39
	≥ 80	51/42	1.07	0.63–1.84
			*p* = 0.596	
Any type	0	23/33	1.00	—
	1–20	60/135	0.81	0.40–1.68
	21–69	91/105	1.62	0.80–3.31
	70–184	94/102	1.38	0.67–2.82
	≥ 185	108/88	1.62	0.79–3.32
			*p* = 0.004	

a Adjusted for age at index date, family history of hematologic cancer, college education, surrogate status and year of interview, frequencies of use of pain-relieving drugs and of cortisone injections, history of eczema/hives, and history of antihistamine use; use of each type of pesticide was adjusted for use of other types of pesticides combined.

b *p*-Values for trend based on natural-log–transformed continuous values.

**Table 3:** ORs and 95% CIs for NHL associated with selected home pesticides by application type.

		Applied by self			Indoor application by others			Outdoor application by others		
Pesticide type	Quartile*^a^*	No. of cases/controls	OR*^b^*	95% CI	No. of cases/controls	OR	95% CI	No. of cases/controls	OR	95% CI
Insecticides for flying	1	42/48	1.60	0.92–2.77	28/40	1.10	0.59–2.02	27/51	0.68	0.37–1.28
bugs or foggers	2	39/52	1.09	0.62–1.91	13/26	0.95	0.42–2.12	37/54	1.07	0.62–1.84
	3	48/43	1.73	0.97–3.03	29/29	1.76	0.90–3.42	43/42	1.56	0.88–2.78
	4	46/44	0.97	0.55–1.71	28/27	1.28	0.65–2.52	53/32	2.37	1.32–4.24
			*p**^c^* = 0.653			*p* = 0.149			*p* = 0.005	
Insecticides for	1	35/51	0.92	0.53–1.61	33/42	1.06	0.59–1.90	25/27	1.16	0.60–2.26
crawling bugs	2	36/50	0.82	0.46–1.44	32/44	1.16	0.65–2.08	25/27	1.12	0.57–2.20
	3	48/40	1.65	0.94–2.88	38/39	1.14	0.62–2.07	25/27	0.87	0.42–1.78
	4	42/44	1.27	0.72–2.24	38/36	1.52	0.84–2.75	31/21	1.69	0.85–3.38
			*p* = 0.098			*p* = 0.327			*p* = 0.205	
Any type	1	55/114	0.88	0.42–1.83	44/59	1.26	0.58–2.76	54/86	1.13	0.54–2.38
	2	76/93	1.36	0.66–2.80	45/71	1.35	0.63–2.90	66/74	1.58	0.75–3.34
	3	84/86	1.51	0.73–3.12	54/56	1.53	0.70–3.32	69/72	1.44	0.69–3.04
	4	92/77	1.64	0.79–3.40	59/51	1.68	0.77–3.67	77/63	1.58	0.75–3.32
			*p* = 0.012			*p* = 0.141			*p* = 0.177	

a Quartile cutoff points for self, indoor by others, and outdoor by others are, respectively, 1–3, 4–15, 16–75, ≥ 76; 1, 2–3, 4–21, ≥ 22; and 1–2, 3–9, 10–48, ≥ 49 for insecticides for flying bugs/foggers; 1–3, 4–13, 14–41, ≥ 42; 1, 2–8, 9–24, ≥ 25; and 1, 2–8, 9–20, ≥ 21 for insecticides for crawling bugs; and 1–8, 9–36, 37–100, ≥ 101; 1–2, 3–9, 10–35, ≥ 36; and 1–6, 7–27, 28–80, ≥ 81 for any type.

b Adjusted for age at index date, family history of hematologic cancer, college education, surrogate status and year of interview, frequencies of use of pain-relieving drugs and of cortisone injections, history of eczema/hives, and history of antihistamine use, in comparison with a common reference group of subjects with no exposure to a given pesticide group through any application methods. Use of each type of pesticide was adjusted for use of other types of pesticides combined.

c *p*-Values for trend based on natural-log–transformed continuous values including level 0 (reference group with no exposure through any application methods).

**Table 4:** ORs*^a^* and 95% CIs for NHL associated with occupational and home pesticide exposure by type of NHL.

	Level of pesticide exposure										
	0	1			2			3			
Exposure type, NHL cell type, and grade	No. of cases	No. of cases	OR	95% CI	No. of cases	OR	95% CI	No. of cases	OR	95% CI	*p*-Value for trend*^b^*
At job*^c^*											
B-cell	238	32	1.06	0.61–1.82	24	2.48	1.21–5.08	24	1.77	0.90–3.48	0.014
T-cell	17	3	2.94	0.61–14.03	3	18.20	3.47–95.44	2	1.79	0.25–12.77	0.005
Low	107	16	1.03	0.52–2.04	17	3.99	1.80–8.80	12	1.80	0.79–4.11	0.007
Intermediate	151	16	1.04	0.53–2.05	10	1.64	0.67–4.04	10	1.34	0.56–3.20	0.276
High	14	4	3.07	0.79–11.98	2	7.27	1.31–40.40	5	6.11	1.46–25.57	< 0.001
No. of controls	371	51			20			20			
At home*^d^*											
B-cell	73	80	1.82	1.16–2.86	79	1.52	0.96–2.40	90	1.76	1.11–2.81	0.014
T-cell	5	5	4.22	0.88–20.25	3	1.56	0.27–8.93	12	3.58	0.83–15.42	0.077
Low	35	34	1.53	0.86–2.73	41	1.44	0.82–2.54	44	1.49	0.83–2.66	0.143
Intermediate	45	49	2.03	1.16–3.55	44	1.64	0.92–2.93	51	1.98	1.11–3.52	0.026
High	2	6	9.90	1.49–65.77	5	6.25	0.92–42.72	12	15.02	2.47–91.29	0.002
No. of controls	168	105			102			88			

a Adjusted for age at index date, family history of hematologic cancer, college education, surrogate status and year of interview, frequencies of use of pain-relieving drugs and of cortisone injections, history of eczema/hives, and history of antihistamine use.

b *p*-Values for trend based on natural-log–transformed continuous values.

c Total number of years at job with pesticide exposure, defined as follows: 0, none; 1, < 10 years; 2, 10–17.9 years; 3, ≥ 18 years.

d Cumulative number of uses of any home pesticides, defined as follows: 0, 0–20; 1, 21–69; 2, 70–184; 3, ≥ 185.
